# Translating GWAS Findings to Novel Therapeutic Targets for Coronary Artery Disease

**DOI:** 10.3389/fcvm.2018.00056

**Published:** 2018-05-30

**Authors:** Le Shu, Montgomery Blencowe, Xia Yang

**Affiliations:** ^1^Department of Integrative Biology and Physiology, University of California, Los Angeles, Los Angeles, CA, United States; ^2^Molecular, Cellular, and Integrative Physiology Interdepartmental Program, University of California, Los Angeles, Los Angeles, CA, United States; ^3^Bioinformatics Interdepartmental Program, University of California, Los Angeles, Los Angeles, CA, United States; ^4^Institute for Quantitative and Computational Biosciences, University of California, Los Angeles, Los Angeles, CA, United States; ^5^Molecular Biology Institute, University of California, Los Angeles, Los Angeles, CA, United States

**Keywords:** genome-wide association study, coronary artery disease, drug targets, multi-omics, functional genomics, networks

## Abstract

The success of genome-wide association studies (GWAS) has significantly advanced our understanding of the etiology of coronary artery disease (CAD) and opens new opportunities to reinvigorate the stalling CAD drug development. However, there exists remarkable disconnection between the CAD GWAS findings and commercialized drugs. While this could implicate major untapped translational and therapeutic potentials in CAD GWAS, it also brings forward extensive technical challenges. In this review we summarize the motivation to leverage GWAS for drug discovery, outline the critical bottlenecks in the field, and highlight several promising strategies such as functional genomics and network-based approaches to enhance the translational value of CAD GWAS findings in driving novel therapeutics

## Introduction

Coronary artery disease (CAD) is a leading cause of mortality worldwide (1). CAD is well recognized as a complex disease with both genetic and environmental contributions (2). The heritability of CAD is estimated to be 40–50% (3), and the genetics of CAD plays an indispensable role in unraveling the pathogenic processes and ultimately facilitating the discovery of novel therapeutics. In the past decade, our understanding of the genetic architecture and mechanistic underpinnings for CAD has been substantially accelerated and broadened, primarily attributable to the successful global collaborative efforts in large-scale human genome-wide association studies (GWAS). These efforts have helped reveal hundreds of novel genetic variants demonstrating significant associations with CAD.

**Figure 1:**
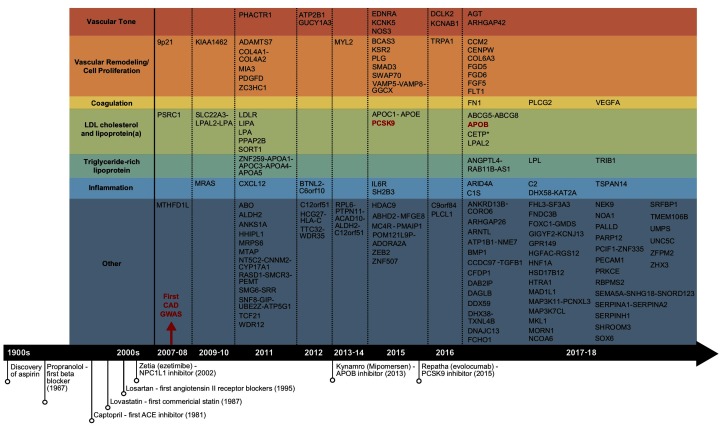
Summary of the reported CAD GWAS loci and important CAD drug discoveries. Candidate genes under GWAS loci identified to date were retrieved from the reported genes column in GWAS Catalog, organized by year. Only one candidate gene per locus was shown. GWAS loci that overlap with the targets of commercialized drugs were shown in red.

In contrast to the gratifying successes of GWAS, the development of CAD drugs has stagnated over the past decades, especially when compared to other therapeutic areas (4). What is particularly concerning is the fact that the drug development effort has been primarily concentrated on correcting previously established CAD risk factors such as lipid levels, coagulation factors, and hypertension, instead of targeting novel pathways revealed from recent studies (Figure 1). This decoupling between mechanistic discovery studies and drug development is striking. Therefore, it is of critical importance to form strategies that leverage the recent genetic discoveries from GWAS and other relevant efforts such as multi-dimensional data integration and systems genetics to allow for efficient identification of novel and reliable CAD drug targets. In this review, we summarize the state of CAD GWAS discovery, delineate the significant challenges of translating GWAS to drug targets, discuss successful examples of GWAS driven CAD drug target discovery, and outline promising strategies to further catalyze the translation of CAD GWAS into novel therapeutic options.

## GWAS Discovery for CAD and Its Implications for Drug Target Discovery

The completion of the human genome project, the rapid declining cost of genome sequencing, the rising feasibility of global multi-group collaborations, and the increasing accessibility of shared data repositories have collectively fueled the explosion of genetic studies of CAD, particularly GWAS. GWAS were typically designed to profile common known variants, often defined as variants with allele frequency ≥ 0.5% (5), on pre-designed microarrays containing primarily single nucleotide polymorphisms (SNPs). Since the first CAD GWAS in 2007 (6), over 18 GWAS studies have been carried out in the past decade, with the most recent and largest study involving 34,541 cases and 261,984 controls (7). These studies revealed a total of 163 genetic loci linked to CAD (8) (Figure 1), explaining 30–40% of CAD heritability (7, 9, 10). The swift pace of GWAS has greatly facilitated the comprehensive construction of the CAD genetic landscape, and has led to rapid accumulation of potential causal variants and genes.

Overall, GWAS have played a key role in not only confirming classic CAD risk factors such as LDL cholesterol, hypertension, and coagulation, but also highlighting the causal roles of cellular proliferation and adhesion, extracellular matrix, and inflammation (Figure 1), which are processes related to the endothelial and smooth muscle cells in the vascular wall and the immune system (3, 11). Unfortunately, to date no novel CAD GWAS genes beyond a few involved in classic risk factors have been established as viable drug targets for CAD, a pattern that resonates for GWAS of most complex traits (Figure 1) (12). The disconnection between CAD GWAS findings and treatment targets is disappointing and has been criticized, but could also implicate major untapped opportunities (13). In particular, the causal variants and genes involved in the new causal pathways informed by GWAS have been encouraging early stage advances in uncovering novel therapeutic options targeting the vascular wall components, cell proliferation, and inflammation. For example, the *ADAMTS7* loci, coding for a metalloproteinase with thrombospondin motifs 7, was implicated for atherosclerotic progression through smooth muscle cell migration, a mechanism independent of classic CAD risk factors (14). Upon confirmation of its causal role in affecting atherosclerosis occurrence *in vivo* (15), development of the ADAMTS7 pharmacophore has progressed towards establishing inhibitors via virtual screening (16). Tocilizumab, an anti-inflammatory agent blocking interleukin-6, was found to improve endothelial function (17). Antibodies targeting CD47, a key anti-phagocytic and tumorigenic molecule, were also shown to ameliorate atherosclerosis by stimulating efferocytosis (18).

Despite the potential promises, several factors could have complicated the extraction of therapeutic value from GWAS. First, the functional regulatory circuits from most variants to disease outcomes remain elusive. This is reflected by both the difficulty in pinpointing the causal variants and the corresponding target genes, especially for variants located in non-coding regions. In fact, the exact effector genes and functions for over 50% of the CAD GWAS loci are unclear. For example, the 9p21 locus was the strongest CAD locus but is located in a gene desert (6, 19, 20). Multiple follow-up studies have suggested several effectors for this locus, including the non-coding RNA ANRIL (21), *CDKN2A*/*CDKN2B* (22, 23), and interferon-gamma signaling (24). However, the detailed mechanism is still under debate after a decade of research (25). Moreover, even if a CAD variant is located within a gene-rich region, the most adjacent gene(s) may not be the functional candidate (26). Second, even if the candidate genes can be unequivocally determined, the functions of the genes are not necessarily well established, and extensive functional studies are required to derive a mechanistic understanding of how the candidate genes lead to CAD risks. Third, most common variants only confer weak to moderate CAD risk (<20% change in risk), most likely due to evolutionary pressure which selects against non-synonymous SNPs in disease genes involved in key physiological processes (12, 27–30). The prevalence of moderate/weak effect sizes of CAD risk variants makes prioritization of drug targets difficult. Lastly, it has been suspected that the top CAD risk variants identified so far predominantly inform on genes active in the early and slow phase of CAD development, whereas variants affecting late and rapid CAD phases tend to be missed by GWAS as these are likely more dependent on specific contexts such as particular environmental exposures or inflammatory states that are poorly controlled in most GWAS (31). Indeed, a recent study of Crohn’s disease that focuses on disease course or prognosis using a within-cases design revealed loci that are completely different from those derived from case-control studies (32). This is also likely the case for CAD. Therefore, drug targets derived from CAD GWAS findings may not carry the expected efficacy to counteract CAD progression.

## Strategies to Fast-Forward the Translation of GWAS to Treatment Targets

**Figure 2:**
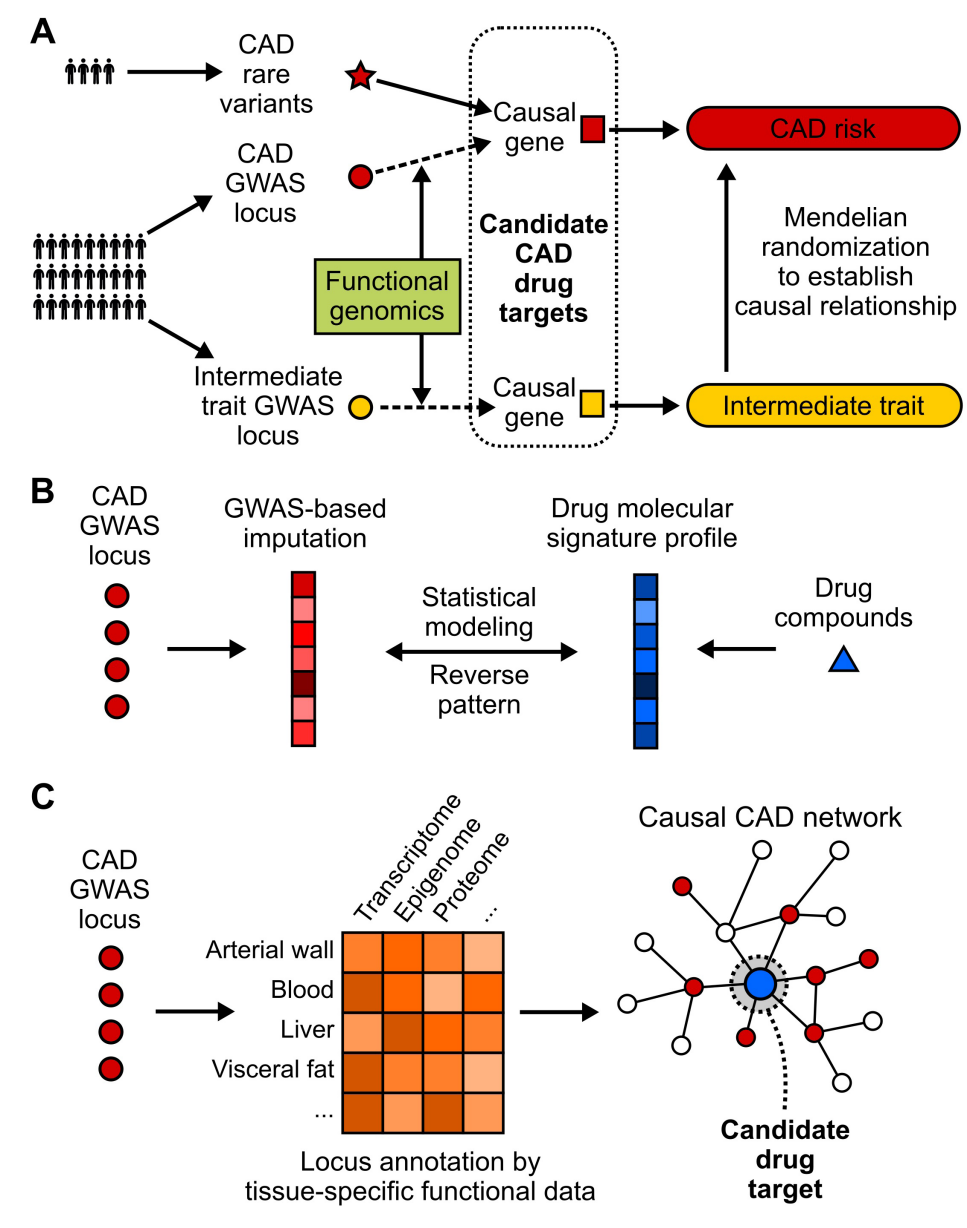
Strategies to translate CAD GWAS into drug targets. **(A)** Identification of CAD causal genes as candidate drug targets by incorporating functional genomics, rare variants and Mendelian randomization. Loss-of-function rare variants can be linked to downstream genes. The connection between common variants and causal genes usually requires integration of functional genomics data. Mendelian randomization can further filter the drug target selection pool by incorporating causal intermediate traits. **(B)** A “target-less” approach to reposition existing drug compounds for CAD by evaluating the existence of opposite patterns between drug molecular profiles and GWAS imputed molecular profiles of disease. **(C)** Network-based approaches that model CAD GWAS data along with other omics data from CAD relevant tissues or cell types in the context of gene networks, which have the power to pinpoint key network regulators as candidate drug targets with more potent effects.

To bypass the challenges facing the translation of GWAS findings to therapeutic targets as outlined above, a number of strategies have been designed and attempted. These efforts mainly focus on integrating GWAS hits with other data types that help inform on the functions of candidate genes, pathways, and networks, narrow down and prioritize the causal candidates, and leverage the matching patterns between disease mechanisms and molecular patterns of drugs (Figure 2).

### Use of Rare Variant Association Studies to Prioritize Targets

As discussed above, common variants uncovered from GWAS studies are numerous in number while carrying weak to subtle effect sizes, making it challenging to prioritize viable targets. Rare genetic variants (frequencies lower than 0.5%) that are associated with diseases, on the other hand, usually exhibit stronger perturbations in gene functions and are under stronger evolutionary pressure. Therefore, rare variants, especially those leading to loss-of-function, provide a natural setting mimicking human knockout cases to assess phenotypic and clinical consequences of variants, and their power in informing causal disease genes and drug targets has been long recognized (Figure 2A) (33, 34). Aggregation of rare mutations in 10 genes, including *APOA5* (35), *APOC3* (36), *ASGR1* (37), *ANGPTL3* (38), *ANGPTL4* (39), *LPA* (40), *LDLR* (35), *LPL* (41), *NPC1L1* (42), and *PCSK9* (43), has been linked to CAD risk through whole exome or whole genome sequencing-based studies. Out of the 10 genes, 5 (*ANGPTL4, APOC3, LPA, NPC1L1, PCSK9)* have been explored as drug targets for CAD (3). To date, the main success of this approach lies in the approval of PCSK9 antibodies by the FDA. Carriers with inactivating mutations on *PCSK9* were found to have markedly lower LDL cholesterol level and CAD risk, which led to the discovery of two FDA-approved monoclonal antibodies, Alirocumab and Evolucumab. However, the potential of drug discovery using CAD rare variants is also limited by both the small number of robust rare variants found so far, and their low cumulative contribution to CAD risk in the general population (9). Additionally, most of them are involved in the previously established pathways rather than novel mechanisms. Nevertheless, these rare variants provide compelling causal inference of the downstream genes and pathways in CAD pathogenesis, and they are more likely to be specific to a disease (broad effects could be detrimental in human knockouts) and have safer profiles, key components for success as drug targets. Future GWAS will likely evolve from SNP array design to whole genome sequencing to profile both common and rare variants (44), thus further expanding the pool of loss-of-function variants for drug target selection. Some of the novel rare variants may inform on novel causal mechanisms not captured by common variants, or converge on genes and pathways already informed by common variants thus serving to enhance the causal inference at a more functional level.

### Functional Genomics to Identify and Prioritize Causal GWAS Genes

In contrast to rare coding mutations whose target genes and downstream mechanisms can be more readily uncovered through traditional functional studies, identifying the causal genes that are responsible for the observed link between GWAS risk variants and CAD is not an easy task. It is estimated that two thirds of the predicted target genes of GWAS locus are not the closest by proximity (45, 46), thus traditional proximity-based locus mapping could introduce false interpretations that bias drug target selection. This challenge can be substantially alleviated by functional genomics tools that explore potential mechanisms linking causal variants to biological phenotypes (Figure 2A) (44). Supported by next-generation sequencing, typical functional molecular traits that may be characterized include expression quantitative trait locus (eQTL), non-coding RNA, transcription factor binding sites, epigenetic modification and chromatin interaction (26). The advance of gene editing technologies such as CRISPR/Cas has also significantly improved the efficiency of validation experiments (47). Recent functional genomics studies have substantially refined the candidate causal genes for CAD loci such as *SORT1* (48), *TRIB1* (49), *ADAMTS7* (15, 50), and *TCF21* (51, 52). Noteworthy, there have also been integrative functional genomics studies that combined genomics, epigenomics and transcriptomics profiling to prioritize causal variants and affected genes (7, 46, 53). For example, Miller et al. integrated Assay for Transposase Accessible Chromatin (ATAC-seq) and chromatin immunoprecipitation-sequencing (ChIP-seq) to unravel the cis-regulatory mechanisms in human coronary artery smooth muscle cells, and prioritized 64 variants over 7 candidate CAD loci including 9p21.3, *SMAD3, PDGFD, IL6R, BMP1, CCDC97/TGFB1* and* LMOD1* (53). Haitjema et al. also leveraged circular chromosome conformation capture sequencing (4C-seq) with RNA-seq and eQTL to identify 294 novel candidate CAD genes (54). These studies greatly contribute to the accumulation of viable treatment targets for follow-up drug development efforts.

Encouragingly, functional studies following GWAS are being further catalyzed by large-scale community efforts in establishing multi-cell or multi-tissue mapping of regulatory annotations. The advent of publicly available depositories such as GTEx (55), ENCODE (56) and Epigenome Roadmap (57) is gradually removing the hurdle to acquire multi-dimensional data resources necessary for the investigation of complex traits like CAD.

### Mendelian Randomization (MR) to Facilitate Drug Target Selection

Previous successes in drug development for CAD have testified to the effectiveness of modulating intermediate causal risk factors such as circulating cholesterol levels and blood pressure in lowering CAD risk. Therefore, knowing the causal relationship between an intermediate phenotype that correlates with CAD status is of monumental importance as it can help prioritize biomarkers as intervention targets for CAD therapeutics (58, 59) (Figure 2A). The investigation of causal intermediate traits for CAD can be facilitated by MR, which utilizes genetic variants as instrumental variables to assess the causal relationship between exposure (e.g., LDL cholesterol, HDL cholesterol, weight-hip ratio) and outcome (CAD occurrence) (60). We are seeing both successful and ongoing efforts in developing drugs modulating LDL cholesterol, triglyceride-rich lipoproteins and lipoprotein (a) (3), whose causal relationships with CAD have been robustly verified in MR studies (61–63). On the contrary, MR studies revealed inconsistent relationship between HDL cholesterol and CAD (64–66). In concordance with this lack of robust support for the causality of HDL in CAD, substantial obstacles have been met during the development of inhibitors for CETP (cholesteryl ester transfer protein), a gene harboring several loci associated with HDL cholesterol level (67). Three commercial CETP inhibitors, Dalcetrapib, Obicetrapib and Anacetrapib, all failed to achieve clinical efficacy during phase III clinical trials and were discontinued (68).

In addition to the well explored causal pathways such as cholesterol and blood pressure regulation, MR studies have informed several additional causal intermediate phenotypes, such as inflammation (69), uric acid (70), and iron status (71), that could serve as targets for future CAD drug development. By utilizing both summary-level GWAS statistics and UK Biobank data, a recent MR study demonstrated the causal association of waist-to-hip ratio adjusted for body mass index with coronary heart disease, thus providing new opportunities of intervening CAD risk by reducing abdominal obesity (72).

### GWAS-Based “Target-Free” Drug Repositioning

Drug repurposing approaches could leverage known drugs used for other diseases that target the newly uncovered CAD causal genes and pathways to counteract CAD. For example, better understanding of CAD pathways involved in inflammation and cell cycle has promoted the repurposing of drugs targeting diseases such as rheumatoid arthritis (17) and cancer (18). On the other hand, given the challenging nature of identifying both the causal genes from GWAS and matching it with the target of drug compounds, “target-free” approaches have been developed which require no prior knowledge of targets for either drugs or GWAS variants and can simultaneously take many genetic loci into consideration (73) (Figure 2B). The fundamental concept behind these approaches is to impute gene expression profiles from GWAS summary statistics, compare the expression patterns against gene expression profiles of drugs, then prioritize top drug candidates whose profiles show reverse patterns compared with GWAS-imputed signatures. This approach is especially useful for repositioning existing drugs whose chemical properties and molecular responses have been well characterized and made accessible from public data repositories such as CMap (74) and its successor, the L1000 platform (75), as well as other chemoinformatic resources (76, 77).

To facilitate such efforts, the work by Gamazon et al. represents one of the first transcriptome imputation pipelines where disease relevant gene expression is estimated from a tissue-dependent model trained with personal genotype data and reference transcriptome (78). Gusev et al. and So et al. further developed summary GWAS statistics based transcriptome imputation methods, which relieved the requirement for individual genotype data (73, 79). In addition, inferring gene expression changes from GWAS enables researchers to assess transcriptome-wide associations with CAD that could yield novel candidate genes for functional and therapeutic investigation (45, 79). Although direct application of the “target-free” approaches for CAD is still under-explored, a computational framework has been developed to reposition existing drugs for psychiatry (73). The framework, built on a GWAS-based transcriptome imputation pipeline named MetaXcan (80), first imputed the gene expression profiles of 10 brain regions for 7 psychiatric disorders based on GWAS and reference transcriptome data from GTEx (55). This disease transcriptome information was then used to match with drug-induced gene expression profiles from the CMap database (74) to prioritize drugs that showed opposite gene expression patterns compared to the disease patterns. These platforms are potentially translatable to CAD.

### Network-Based Drug Discovery Approaches

The success of GWAS-driven drug target identification heavily relies on the fundamental assumption of how genetic risk variants eventually contribute to disease phenotype. An “omnigenic” model for the genetics of complex traits has been recently proposed (30). This provocative model objects the common belief that risk variants drive disease etiology through functional clustering in biological pathways, and emphasizes that all genes in disease-relevant cells could affect core disease processes through the coordination of gene regulatory networks.

Motivated by the gene network hypothesis, the CAD field has been actively investing on the development and application of systems genetics frameworks that integrate genetics and other data dimensions in the context of network topology to help prioritize candidate CAD genes (Figure 2C) (27–29, 81–84). The implementation of network-based target identification strategies poses several unique advantages over other methods. First, gene networks have the potential to comprehensively map the regulatory circuits under physiological or pathological conditions, thus improving the biological relevance of predicted targets. Second, gene networks serve as a natural platform for data integration, where GWAS and information from other omics space can be collectively leveraged to pinpoint network hotspots where key perturbation events likely happen. Third, gene networks enable the identification of essential disease genes, which is unlikely to tolerate high frequency loss-of-function variation at the population level and to be discovered by GWAS (85). Several methods have been developed to find network essential genes, or key drivers, by considering both network topology and external disease signatures (27, 81, 86). The validity of the predicted key drivers in driving CAD relevant traits has been well supported (27, 29, 82), and the key drivers have the potential to serve as novel drug targets with strong therapeutic effects due to their central importance in regulating the disease networks. For instance, Zhao et al. recently prioritized CAD key drivers and proposed plausible targets using network approaches (28). Extensive *in vitro* and *in vivo* gene perturbation experiments are required to evaluate the feasibility of using key driver genes as drug targets. If proven valid, network-based discoveries could provide exciting opportunities to formulate more focused and data-informed hypotheses for downstream therapeutic investigation. Nevertheless, it is important to caution that modulation of network key drivers may result in a lack of specificity and increase the risks for side effects due to their broad impact on numerous network genes.

One critical challenge for network-based CAD drug discovery is the availability of high-quality gene networks from CAD relevant cells, tissues, and subjects. Many existing networks are literature-based and lack tissue/cell specificity. Even for data-driven networks, data collection bias exists. For example, human network construction usually requires large numbers of clinical samples that are difficult to acquire, especially for samples from internal tissues. A major breakthrough is the establishment of the STARNET networks involving tissue-specific data from ~600 CAD patients (87). This resource, in combination with other networks generated from mouse models or non-disease human subjects, is invaluable for future CAD network studies. Coordinated efforts by the research community are needed to enhance the coverage of data-driven networks from CAD relevant tissues and cell types.

## Conclusions and Future Directions

GWAS has been highly successful in elucidating the genetic architecture of CAD and driving the discovery of novel biology. While confirming the genes and pathways targeted by classic CAD treatments, GWAS opens doors to a vast number of under-recognized candidates where future CAD drugs could originate. The field of GWAS-driven drug discovery is still at its infancy, and significant challenges remain. However, it is encouraging that numerous methodological advances have been made to address the bottlenecks, and application of these approaches is expected to facilitate future translational research in CAD.

Here we anticipate the following future directions that will help further advance the field. First, there is a need for broader collaboration to conduct large-scale functional genomics studies in human tissues and cell types that implement cutting-edge high-throughput profiling technologies over multiple omics to map the tissue- and cell-type specific regulatory circuit of GWAS loci. In particular, application of cell-type specific analyses at multi-omics levels will help address the functional heterogeneity in CAD relevant tissues, which will lead to refined understanding of disease etiology and lay a solid foundation for more accurate prediction of drugs that can counteract the specific pathogenic processes in the right cell types and tissues (88). The recent launch of the Human Cell Atlas project represents one of the first stepping stones towards this direction (89). Second, more efficient platforms are needed to facilitate sharing of summary-level GWAS data as well as databases and data repositories that curate multi-omics functional information. For example, in the neurological disorder field, there are emerging efforts like CommonMind (http://commonmind.org), PsychENCODE (90) and BrainSeq (91). Similar coordinated efforts by the CAD community will accelerate identification of CAD drug targets. Third, the translational value of GWAS data can be better exploited by the development of novel analytical pipelines that integrate multi-dimensional data from animals, humans, and chemoinformatic databases. Some of the recently developed analytical pipelines can integrate GWAS and functional genomics data for target prediction, and are directly applicable to CAD (45, 79, 81, 92). Additional pipelines that couple disease datasets with drug footprints in a gene network framework will facilitate the identification of network regulators and pathways that can be accurately targeted. Lastly, gradual transition from initial target screening to GWAS-guided experimental validation of the predicted targets using a combination of *in vitro*, *in vivo*, and *in silico* methods will further the translational path.

## Author Contributions

LS, MB and XY drafted and edited the manuscript.

## Conflict of Interest Statement

The authors declare that the research was conducted in the absence of any commercial or financial relationships that could be construed as a potential conflict of interest.
